# The role of multimodality imaging in diagnosing acute perimyocarditis secondary to Crohn’s disease

**DOI:** 10.1186/s12872-021-02232-x

**Published:** 2021-09-10

**Authors:** Hawani Sasmaya Prameswari, Iswaree Devi Balakrishnan, Chun Yuan Khoo, Loon Yee Teo, Lihua Laura Chan, Choon Ta Ng

**Affiliations:** 1 Department of Cardiology, Hasan Sadikin General Hospital, Universitas Padjadjaran, Jalan Prof. Eyckman No.38 , Bandung, West Java 40161 Indonesia; 2 Department of Cardiology, National Heart Centre, Singapore, Singapore

**Keywords:** Case report, Crohn’s disease, Acute perimyocarditis, ^18^FDG PET—scan

## Abstract

**Background:**

Acute perimyocarditis is a rare extra-intestinal manifestation in Crohn’s disease which required multimodality imaging to confirm the diagnosis. Here we present a case of acute perimyocarditis as the first presentation of Crohn’s disease. To date, this is the first case presentation reporting the use of ^18^F-FDG PET/CT Scan for diagnosing such condition.

**Case presentation:**

A 25-year-old male presented to our hospital with severe persistent pleuritic sharp left-sided chest pain. This was his second hospital admission in the past 4 months for chest pain and diarrhea. At the first hospitalization, he was diagnosed with viral perimyocarditis and irritable bowel syndrome. Laboratory findings, electrocardiogram, and cardiac magnetic resonance imaging results confirm the diagnostic of perimyocarditis. Virology, bacteriology, parasitology, and autoimmune evaluations were unremarkable. Colonoscopy, colorectal biopsy, and ^18^FGD PET findings confirmed manifestation of perimyocarditis, Crohn’s disease, and negative for sarcoidosis.

**Conclusions:**

Looking at the overall clinical picture and investigation results of colonoscopy, colorectal biopsy findings, as well as multi-modality imaging with echocardiography, ^18^FDG PET—scan and CMRI, the patient was diagnosed to have perimyocarditis attending Chron’s disease flare up as a rare extra-intestinal manifestation.

## Background

Crohn’s disease is an idiopathic inflammatory bowel disease (IBD) that can affect any part of gastrointestinal tract. Extra-intestinal manifestations involving the musculoskeletal, dermatologic, hepatobiliary, ocular, renal, and pulmonary system have been reported in IBD patients. Cardiac involvement such as perimyocarditis is, however, extremely rare, with reported prevalence of 0.04 %, but carries high risk of mortality [1, 2]. Acute myocarditis can occur during IBD flare up or immunosuppressant therapy [3, 4]. We present a case of acute perimyocarditis as the first presentation of Crohn’s disease, using multimodality imaging modalities such as CMRI and whole body ^18^FDG PET/CT scan.

## Case presentation

A 25-year-old male was presented to our hospital with severe persistent pleuritic sharp left-sided chest pain. He described recurrent sharp chest discomfort which was not aggravated by breathing or positional change. He did not have a heart failure condition, shortness of breath, orthopnoea, nor paroxysmal nocturnal dyspnoea. Moreover, he did not have any cardiovascular risk factors. Upon further questioning, he reported episodes of bloodless diarrhea associated with mild abdominal pain for the past 4 months. He had no dysphagia, odynophagia, or change in his bowel habits.

This was his second hospital admission in the past 4 months for chest pain and diarrhea. At the first hospitalization, he was diagnosed with viral perimyocarditis and irritable bowel syndrome. After his discharge, he continued experiencing intermittent chest discomfort 2–3 times a month.

**Fig. 1:**
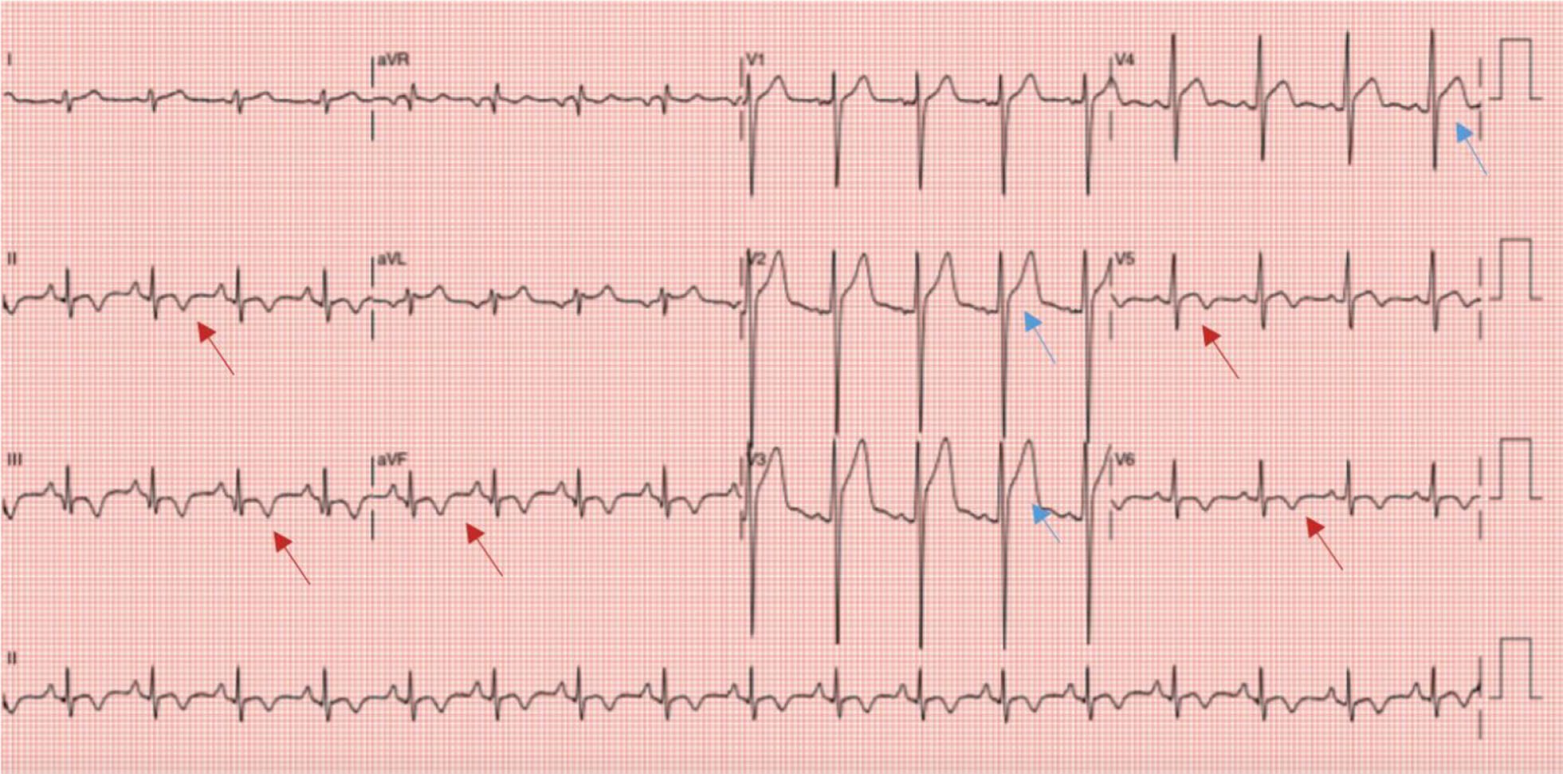
Electrocardiogram of patient on presentation. Electrocardiogram shows sinus tachycardia with anterior concave ST-segment elevation (blue arrows) and T-wave inversion in inferolateral leads (red arrows)

Physical examination revealed a clinically stable and non-toxic patient. Initial laboratory findings demonstrated an increased white blood cell count of 14.100/mm^3^, as well as elevated cardiac biomarkers (Troponin T of 278 ng/L and CKMB of 9 µg/L). An electrocardiogram showed sinus tachycardia with shallow T-wave inversion in the inferolateral leads, and anterior ST-segment elevation (Fig. 1). Trans-thoracic Echocardiography (TTE) revealed normal left ventricular (LV) size with preserved LV function and no pericardial effusions. Computed tomography (CT) coronary angiogram showed normal coronaries, excluding coronary artery disease. Based on these clinical and investigation findings, the impression was that of acute perimyocarditis.

**Fig. 2:**
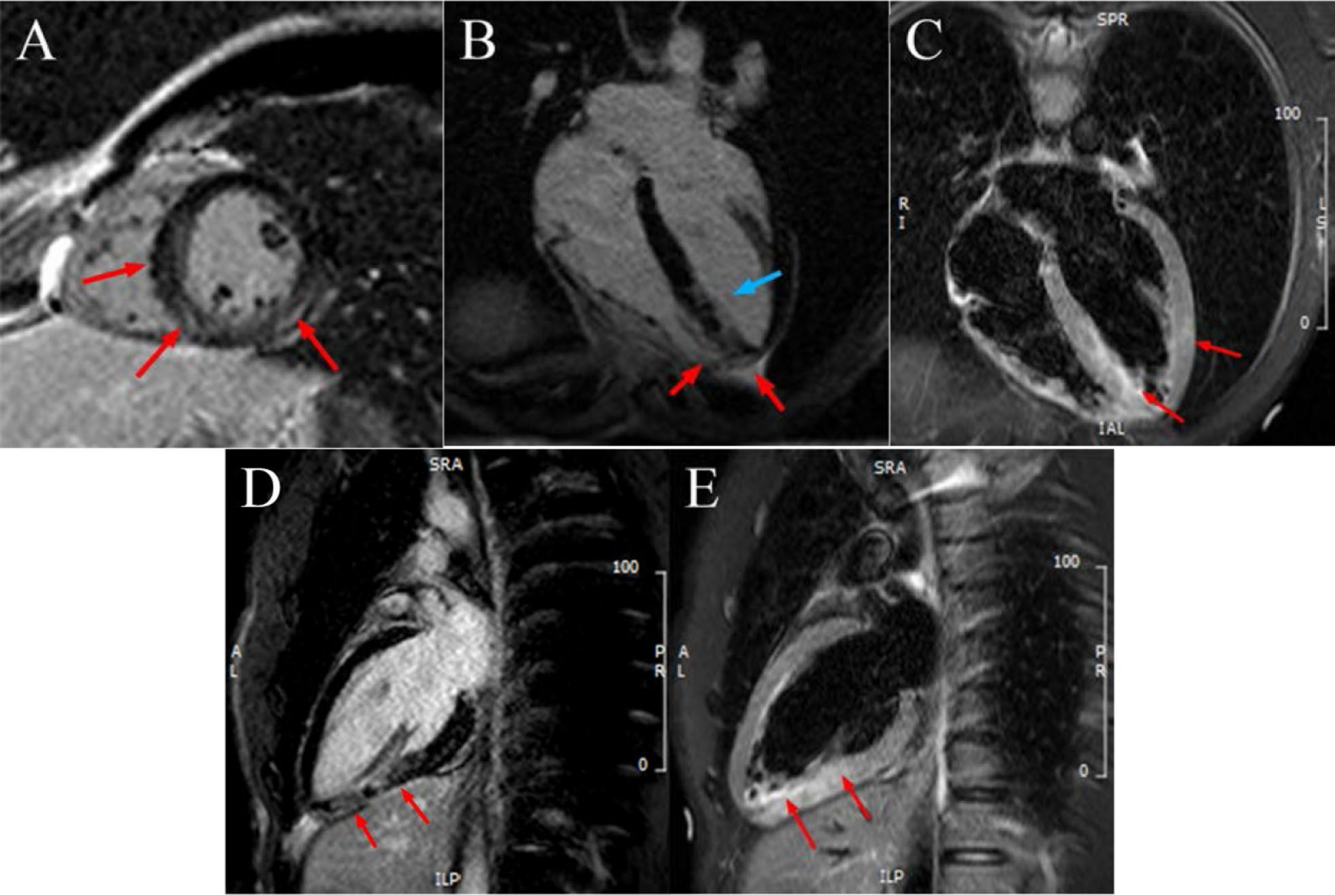
Cardiac MRI of patient. **A** CMR late gadolinium in SAX view showed patchy LGE of the mid to apical inferior and septal segments; and pericardial enhancement in the inferior and inferolateral wall, **B** LGE of the mid to apical inferior and septal segments at 4 chambers, **C** Septal and lateral hyper-enhancement at T2W STIR 4 chambers, **D** LGE of the mid and apical inferior at 2 chambers, **E** Mid and apical inferior hyper-enhancement T2W STIR 2 chambers

Cardiac magnetic resonance imaging (CMRI) demonstrated patchy late gadolinium enhancement (LGE) of the mid to apical inferior and septal segments; and pericardial enhancement in the apical inferolateral and inferior wall. T2 weighted (T2W) imaging showed increased signal intensity in the mid inferior and apical inferior, septal and lateral segments. These findings are suggestive acute perimyocarditis or cardiac sarcoidosis (Fig. 2).

Considering that recurrent onset of acute perimyocarditis is extremely uncommon, other diagnoses, such as cardiac sarcoidosis was considered. In the interim, we performed a comprehensive workup to exclude viral cause of myocarditis. The HIV, Hepatitis B/C, and viral polymerase chain reaction (PCR) comprising parainfluenza, metapneumovirus, rhinovirus A/B/C, human coronavirus, adenovirus, enterovirus, bocavirus, astrovirus, norovirus, rotavirus A, sapovirus, influenza A and B were all negative. Bacteriology and parasitology evaluations were unremarkable. Autoimmune screen [extractable nuclear Antigen Antibody (ENA), Antinuclear Antibody (ANA), and double stranded Deoxyribose Nucleic Acid (ds-DNA) test] was unremarkable.

Further workup of his chronic diarrhea revealed a high stool calprotectin level of 855 µg/g. Stool analyses for ova/parasites/cysts and stool cultures were negative. In view of his chronic diarrhea and elevated calprotectin levels, he was referred to a gastroenterologist for colonoscopy evaluation.

**Fig. 3:**
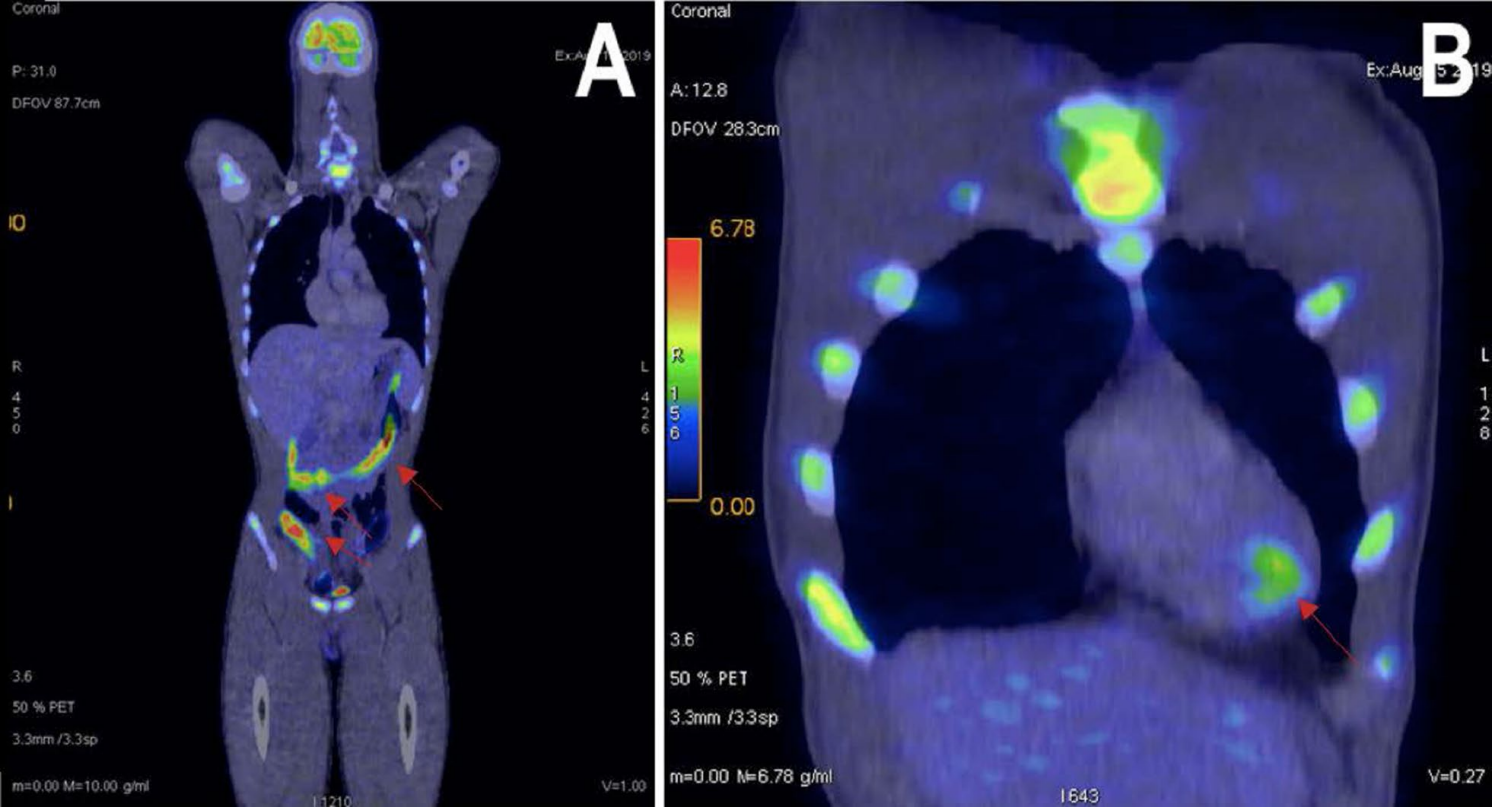
Whole body ^18^FDG PET/CT scan. **A** Diffuse moderate-intensity of FDG uptake by small and large bowels. **B** Mild hypermetabolic focus at apical aspect of interventricular septum

Based on the CMRI findings, both perimyocarditis and sarcoidosis were possible differentials. Therefore, we arranged for a whole-body ^18^Fluorodeoxyglucose Positron Emission Tomography/Computed Tomography (^18^FDG PET/CT) scan. The patient was prepared with a high fat/very low carbohydrate diet 2 meals before scan followed by a fast of at least 4 h to suppress ^18^FDG uptake from normal myocardium. The ^18^FDG PET/CT scan showed a hypermetabolic focus at apical of interventricular septum (maximum Standardized Uptake Value/ SUV max = 3.8), favoring myocarditis rather than cardiac sarcoidosis. In addition, the presence of diffuse moderate-intensity of FDG uptake by small and large bowels (SUV max = 12.2) on the ^18^FDG PET—scan (Fig. 3) further reinforced our initial suspicion of an active inflammatory process in the intestinal tract leading to acute perimyocarditis.

Colonoscopy showed colitis at the rectum, recto-sigmoid, colon and caecum with Crohn’s disease appearance at the terminal ileum. Colorectal biopsy revealed acute-on-chronic inflammation with non-caseating granulomas, consistent with Crohn’s disease. Tuberculosis (TB) of the gut was also excluded by a negative TB DNA amplification test, QuantiFERON®-TB test, Acid Fast Bacteria (AFB) culture and smear.

Looking at the overall clinical picture and investigation results of colonoscopy, colorectal biopsy findings, as well as the ^18^FDG PET—scan and CMRI results, the patient was diagnosed with perimyocarditis secondary to Crohn's disease flare up which is as a rare extra-intestinal manifestation.

He was commenced on immunosuppressant therapy with oral corticosteroids (prednisolone) and azathioprine with complete resolution of his chest discomfort and diarrhea as well as normalization of the troponin level. He remained well and asymptomatic during his follow-up visit at 6 months.

## Discussion and conclusion

Myocarditis is a rare extra-intestinal manifestation in Crohn’s disease, with a reported prevalence of 0.04 %, and can occur in patients between 20 and 50 years old [1, 5–8]. Patients can present with a variety of symptoms ranging from mild symptoms to cardiogenic shock with high mortality risk [4]. Pericardial involvement is infrequent, manifesting as perimyocarditis [3, 9].

In Crohn’s disease, acute perimyocarditis may occur as a complication of either the disease itself or immunosuppressant therapy [3]. In our patient, acute perimyocarditis developed as the first presentation of Crohn’s disease.

While the pathophysiology of perimyocarditis in Crohn’s disease remains uncertain [2, 5], there are two possible pathogenic mechanisms. Firstly, it may be immune mediated resulting from genetic susceptibility, abnormal self-recognition, and autoantibodies against specific cellular antigens shared by the gastrointestinal tract and myocardium or pericardium [9]. Extra-intestinal cardiac manifestations such as myocarditis occurred mostly during high-inflammation activity episodes of Crohn’s disease [3]. Secondly, perimyocarditis in Crohn’s disease may occur due to adverse effect of the treatment with 5-aminosalicylic acid and its derivatives.[10].

In this patient, with recurrent onset of perimyocarditis, cardiac sarcoidosis was considered a differential diagnosis. Histological findings of non-caseating granulomas can be found in both Crohn’s disease and cardiac sarcoidosis, given that both disorders share a similar immunologic response [11]. Over the past few years, non-invasive imaging modalities have provided a safe and non-invasive alternative method for evaluating myocarditis etiology without the risks of cardiac endomyocardial biopsy. In our case, the whole body ^18^FDG PET scan result has led us to the precise etiology of perimyocarditis which showed a single hypermetabolic focus at the apex of the interventricular septum, and at the large/small bowel FDG uptake. Whereas in cardiac sarcoidosis, ^18^FDG PET—scan will classically demonstrate heterogeneous with high tracer accumulation mainly in the sub epicardial, mid myocardial level, and basal, septal segments of the ventricle. Some cases may also involve the lymphatic system [12]. FDG PET is a superior diagnostic tool for perimyocarditis and cardiac sarcoidosis compared to cardiac MRI alone [13, 14]. In our patient, the use of multi-modality imaging CMRI and FDG PET led us to the diagnosis of Crohn’s diseases with perimyocarditis as an extra-intestinal manifestation. To our knowledge, this is also the first case presentation reporting the use of FGD PET Scan and multimodality imaging in diagnosing acute perimyocarditis due to underlying Crohn’s disease [7, 15–18].

While endomyocardial Biopsy (EMB) remains the gold standard to diagnose myocarditis, it was not performed in this patient, given the absence of hemodynamic instability, bradyarrhythmia (Mobitz II or complete heart block), ventricular arrhythmias, or unexplained new-onset heart failure [19]. Furthermore, in our case, EMB was also limited by sampling errors with consideration of segmental involvement based on CMRI and ^18^FDG PET—scan results [20].

Although IBD and sarcoidosis may have similar cardiac manifestations, the inflammatory process in IBD mainly involves the gastrointestinal tract. Therefore, comprehensive clinical history-taking and the use of multi-modality imaging such as CMRI and ^18^FDG PET would help in confirming the diagnosis.

A comprehensive work up involving multi-modality imaging for autoimmune disorder is pivotal in patient presenting with recurrent perimyocarditis. In our patient, the workup of acute perimyocarditis led to the diagnosis of underlying Crohn’s disease. Due to overlapping pathophysiology in autoimmune disease, a thorough extensive evaluation of clinical manifestations, laboratory, and multi-modality imaging findings is pivotal to making an accurate diagnosis. In our patient, the findings of FDG uptake in small or large bowel on the ^18^FDG PET—scan led to the next appropriate investigation. Colonoscopy showed features of Crohn’s disease at the ileum segment with evidence of acute on chronic non-caseating granulomatous inflammation on colorectal biopsy. Consequently, immunosuppressant therapy with prednisolone and azathioprine was started, leading to complete resolution of perimyocarditis in our patient.

## Data Availability

All relevant data supporting the conclusions of this article are included within the article.

## Abbreviations

^18^FGD PET/CT: ^18^Fluorodeoxyglucose positron emission tomography/computed tomography (^18^FDG PET/CT)
AFB: Acid fast bacteria
ANA: Antinuclear antibody
ds-DNA: Double stranded deoxyribose nucleic acid
CMRI: Cardiac magnetic resonance imaging
CT: Computed tomography
EMB: Endomyocardial biopsy
ENA: Extractable nuclear antigen (ENA)
IBD: Inflammatory Bowel disease
LGE: Late gadolinium enhancement
LV: Left ventricular
PCR: Polymerase chain reaction
SUV max: Maximum standardized uptake value
T2W: T2 weighted
TB: Tuberculosis
TTE: Trans-thoracic echocardiography (TTE)
